# Dose-response association between physical activity and sedentary time categories on ageing biomarkers

**DOI:** 10.1186/s12877-019-1284-y

**Published:** 2019-10-15

**Authors:** Asier Mañas, Borja del Pozo-Cruz, Irene Rodríguez-Gómez, Javier Leal-Martín, José Losa-Reyna, Leocadio Rodríguez-Mañas, Francisco J. García-García, Ignacio Ara

**Affiliations:** 1 GENUD Toledo Research Group, Universidad de Castilla-La Mancha, Avda. Carlos III s/n, 45071 Toledo, Spain; 2 CIBER of Frailty and Healthy Aging (CIBERFES), Madrid, Spain; 3 Motivation and Behaviour Research Program, Institute for Positive Psychology and Education, Faculty of Health Sciences, Australian Catholic University, Sydney, Australia; 4 Geriatric Department, Hospital Virgen del Valle, Toledo, Spain; 5 Geriatric Department, Hospital Universitario de Getafe, Getafe, Spain

**Keywords:** Exercise, Ageing, Functioning and disability, Health behaviour, Lifestyle

## Abstract

**Background:**

Physical activity and sedentary behaviour have been suggested to independently affect a number of health outcomes. To what extent different combinations of physical activity and sedentary behaviour may influence physical function and frailty outcomes in older adults is unknown. The aim of this study was to examine the combination of mutually exclusive categories of accelerometer-measured physical activity and sedentary time on physical function and frailty in older adults.

**Methods:**

771 older adults (54% women; 76.8 ± 4.9 years) from the Toledo Study for Healthy Aging participated in this cross-sectional study. Physical activity and sedentary time were measured by accelerometry. Physically active was defined as meeting current aerobic guidelines for older adults proposed by the World Health Organization. Low sedentary was defined as residing in the lowest quartile of the light physical activity-to-sedentary time ratio. Participants were then classified into one of four mutually exclusive movement patterns: (1) ‘physically active & low sedentary’, (2) ‘physically active & high sedentary’, (3) ‘physically inactive & low sedentary’, and (4) ‘physically inactive & high sedentary’. The Short Physical Performance Battery was used to measure physical function and frailty was assessed using the Frailty Trait Scale.

**Results:**

‘Physically active & low sedentary’ and ‘physically active & high sedentary’ individuals had significantly higher levels of physical function (β = 1.73 and β = 1.30 respectively; all *p* < 0.001) and lower frailty (β = − 13.96 and β = − 8.71 respectively; all *p* < 0.001) compared to ‘physically inactive & high sedentary’ participants. Likewise, ‘physically inactive & low sedentary’ group had significantly lower frailty (β = − 2.50; *p* = 0.05), but significance was not reached for physical function.

**Conclusions:**

We found a dose-response association of the different movement patterns analysed in this study with physical function and frailty. Meeting the physical activity guidelines was associated with the most beneficial physical function and frailty profiles in our sample. Among inactive people, more light intensity relative to sedentary time was associated with better frailty status. These results point out to the possibility of stepwise interventions (i.e. targeting less strenuous activities) to promote successful aging, particularly in inactive older adults.

## Background

There is compiling evidence showing the benefits of regular physical activity to improve physical functioning and reduce frailty among the elderly [1, 2]. Consequently, physical activity, particularly more strenuous activity is now routinely recommended in the clinical management of frailty [2, 3]. Blodgett et al. [4] and Manas et al. [5] have shown that moderate-to-vigorous physical activity (MVPA) is inversely associated with frailty and adverse health outcomes in middle-age (≥50 years) and older adults (≥65 years), respectively. However, few older adults meet the physical activity recommendations (i.e., 150 min of moderate intensity aerobic activity, 75 min of vigorous intensity aerobic activity, or an equivalent combination, in 10-min bouts [6]). In fact, previous research has found that older adults spend between 8 and 12 h of their waking day sedentary [7]. Sedentary behaviours, such as TV viewing, motorized transport, or leisure-time sitting, have been shown to contribute to adverse health outcomes in older people, including lower levels of physical functioning and higher levels of frailty [4, 8–10].

Nevertheless, we are far from a complete understanding of the inter-relationships between MVPA and sedentary behaviours and the role they may play on preserving physical function and reducing frailty levels among older adults. Several studies have shown that some people can meet the physical activity recommendations and yet display high levels of sedentary behaviours. The reverse could also be true. Thus, different combinations of behaviours (i.e. ‘physically active and low sedentary’, ‘physically active and high sedentary’, ‘physically inactive and low sedentary’, and ‘physically inactive and high sedentary’) are plausible during waking times. Potentially, these distinct combinations of behaviours may lead to a gradient of health consequences [11, 12]. For example, Bakrania et al. [11] found that physically active adults, even those who spent much of their time on sedentary behaviours, had better cardiometabolic health than those who were inactive. It was also suggested that those individuals with lower sedentary status in the absence of meeting the physical activity recommendations had better cardiometabolic health profile compared to those with higher sedentary status and that were physically inactive (i.e. did not meet the physical activity guidelines). This dose-response pattern has also been confirmed for biological markers and mortality in previous studies [12, 13].

Appreciation of potential physical function and frailty consequences that different combinations of mutually exclusive waking behaviours may have among older adults will be advantageous to target successful public health interventions. For instance, increasing light intensity physical activity could be a feasible approach to improve the physical functioning and reduce the level of frailty of older adults categorize as inactive and high sedentary. Further, if a dose-response exists between the different movement behaviour patterns and physical functioning/frailty in older individuals (i.e., if more active patterns of behaviour are associated with better health profiles), a gradual range of stepwise interventions can be proposed. For example, if someone is sitting in the lowest movement category (i.e., inactive, high sedentary), we could focus on an intervention that targets sedentary behaviour first to move that particular person from inactive, high sedentary to inactive, low sedentary. There are, however, no existing studies analysing the associations between mutually exclusive categories of physical activity and sedentary time with physical function and frailty in older adults. Therefore, the purpose of this study was to examine the combination of mutually exclusive categories of accelerometer-measured physical activity and sedentary time on physical function and frailty in a community-dwelling sample of older adults.

## Methods

### Study design and participants

The current study included a sample of 871 community-dwelling older adults (416 women) from wave 2 (2012 to 2014) and wave 3 (2015 to 2017) of the Toledo Study for Healthy Aging (TSHA) [14]. The methodology of the TSHA study has been described in detail elsewhere [5, 15]. Briefly, the TSHA is a population-based prospective cohort study originally conceived to explore the determinants and consequences of ageing and frailty in older adults from Toledo, Spain. All participants gave their written informed consent prior enrolment. All procedures were approved by the Clinical Research Ethics Committee of the Toledo Hospital and were conducted in accordance with the Declaration of Helsinki for human studies.

### Measurements

#### Frailty status

Frailty was assessed by means of the Frailty Trait Scale (FTS) [16]. The FTS includes 7 domains calculated from 12 items including energy balance and nutrition, assessed using the body mass index, central obesity (waist circumference), unintentional weight loss and serum albumin levels; activity levels, assessed using the total score of the Physical Activity Scale for the Elderly [17]; the nervous system performance, evaluated based on was verbal fluency (estimated by asking the participants to give names of animals during one minute [18]) and balance (Romberg test [19]); the vascular system, measured by the brachial-ankle index done with Doppler ultrasound [20]; weakness, estimated with the grip strength in the dominant arm and the knee extension strength [14]; endurance, assessed by the chair stand test, which measures the number of times that a person stands up in 30 s [21]; and slowness, estimated by calculating the time to walk 3 m at a “normal pace” according to a standard protocol [19]. Scoring is detailed elsewhere [16]. The Total FTS score ranged from 0 (less frailty) to 100 (more frailty).

### Physical Function

The Short Physical Performance Battery (SPPB) was used to assess physical function in this study [19]. Previous studies have shown that low scores on the SPPB have a high predictive value for a wide range of health consequences comprising disability [22], hospitalization [23], and death [24].

The SPPB measures gait speed (8-ft walk), standing balance, and lower extremity strength and endurance (chair rise task). A maximum of 4 points each for the balance, chair stand, and gait speed tests may be awarded, for a score between 0 and 12 (best), in which only integers are allowed [19].

#### Physical activity and sedentary time assessment

Physical activity and sedentary time were assessed via accelerometry (ActiTrainer and ActiGraph wGT3X-BT; ActiGraph, LLC, Pensacola, FL). Participants were instructed to wear an accelerometer on the left hip during waking hours for 7 consecutive days and to remove the accelerometer only before going to bed or for water activities [25]. A valid day was defined as having ≥480 min (≥8 h) of monitor wear, and the study included the results from participants with at least four valid days [26, 27]. Accelerometer cut-points for sedentary time were 0–99 cpm, 100–1951 cpm for light physical activity, 1952–5724 cpm for moderate physical activity, and ≥ 5725 cpm for vigorous physical activity based on previously established cut-points [28]. These cut-off values have been used in previous analyses from the TSHA [5, 15]. In addition, moderate physical activity, vigorous physical activity and MVPA time accumulated in bouts of ≥10 min, allowing for a two-minute exception in the intensity threshold, were also derived. The total minutes in each intensity band were averaged over the number of valid days to estimate the mean time spent in each activity band.

#### Physical activity and sedentary time categories determination

We followed the methods outlined in Bakrania et al. [11] to classify participants in this study into 4 mutually exclusive behavioural categories according to their levels of physical activity and sedentary behaviour. Based on Bakrania et al. [11], and other studies [12, 29], the light physical activity-to-sedentary time ratio was used to classify participants in this study as low sedentary if they resided in the first quartile. Given that most of our sample was expected to be sedentary [7, 15], the remaining participants (i.e. those in quartiles 2, 3, and 4 of light physical activity-to-sedentary time ratio) were classified as high sedentary. MVPA status was classified as ‘physically active’ or ‘physically inactive’ on the basis of whether or not participants met the WHO (World Health Organization) physical activity recommendations for older adults [30]. For this, at least one of these three premises had to be met: accumulate 150 min of moderate physical activity per week over periods of at least 10 min; accumulate 75 min of vigorous physical activity per week over periods of at least 10 min, or accumulate 150 min per week of an equivalent combination of MVPA over periods of at least 10 min.

Based on previous studies [31], four groups of mutually exclusive movement patterns were created: [1] ‘physically active and low sedentary’, [2] ‘physically active and high sedentary’, [3] ‘physically inactive and low sedentary’, and [4] ‘physically inactive and high sedentary’.

#### Confounding variables

Participants were asked about their age, sex and ethnicity. Other socio-demographic variables such as education, income, and marital status were also self-reported in face-to-face interviews as described elsewhere [15].

### Statistical analysis

Analyses were performed using the statistical software SPSS version 24.0 (IBM Corp., Armonk, NY). Participant characteristics of the full sample, stratified by each category, were tabulated. Mean (standard deviation) and frequency (percentage) were provided for continuous and categorical variables, respectively. Ternary plots with the three behaviours were generated to show the distribution of the sample compositions using R statistical system version 3.1.1. To test our hypothesis, a multiple linear regression analysis with the behavioural category as independent variable and frailty or physical function as dependent variable was fitted. Covariates in the model included: age, sex, education, marital status, and income. The ‘physically inactive and high sedentary’ category was selected as the reference category.

Also, the continuous association between time spent in sedentary activities as well as MVPA with the outcomes of interest in the study were explored via regression. The same set of covariates in addition to accelerometer wear time as well as both continuous MVPA time and sedentary status was used.

All analyses were two-sided where *p* ≤ 0.05 was considered to be statistically significant.

## Results

### Descriptive

Of the 871 eligible subjects, 100 participants had insufficient accelerometer wear time so 771 participants were finally included (Table 1).

**Table 1:** Participant characteristics

Characteristics	Sample	‘Physically active & low sedentary’	‘Physically active & high sedentary’	‘Physically inactive & low sedentary’	‘Physically inactive & high sedentary’
	*N* = 771	*n* = 38; 4.9%	*n* = 89; 11.5%	*n* = 154; 20.0%	*n* = 490; 63.6%
Age (years) ^a^	76.8 (4.9)	74.4 (4.0)	74.8 (3.7)	75.9 (4.5)	77.7 (5.1)
Sex ^b^					
Male	355 (46.0)	23 (60.5)	62 (69.7)	50 (32.5)	220 (44.9)
Female	416 (54.0)	15 (39.5)	27 (30.3)	104 (67.5)	270 (55.1)
Education ^b^					
None	487 (63.2)	19 (50.0)	46 (51.7)	97 (63.0)	325 (66.3)
Primary school	169 (21.9)	11 (28.9)	26 (29.2)	39 (25.3)	93 (19.0)
Secundary or more	109 (14.1)	8 (21.1)	17 (19.1)	16 (10.4)	68 (13.9)
Missing ^c^	6 (0.8)	0 (0.0)	0 (0.0)	2 (1.3)	4 (0.8)
Income ^b^					
Low	369 (47.8)	23 (60.5)	45 (50.6)	66 (42.8)	235 (48.0)
Medium	299 (38.8)	11 (29.0)	33 (37.1)	75 (48.7)	180 (36.7)
High	56 (7.3)	3 (7.9)	6 (6.7)	7 (4.6)	40 (8.1)
Missing ^c^	47 (6.1)	1 (2.6)	5 (5.6)	6 (3.9)	35 (7.1)
Marital status ^b^					
Single	42 (5.4)	2 (5.3)	1 (1.1)	9 (5.8)	30 (6.1)
Married	541 (70.2)	28 (73.7)	73 (82.0)	112 (72.7)	328 (66.9)
Widowed	171 (22.2)	7 (18.4)	13 (14.6)	30 (19.5)	121 (24.7)
Divorced/Separated	12 (1.6)	1 (2.6)	2 (2.2)	0 (0.0)	9 (1.8)
Missing ^c^	5 (0.6)	0 (0.0)	0 (0.0)	3 (1.9)	2 (2.4)
Body mass index (kg/m^2^) ^a^	30.3 (4.8)	26.9 (3.8)	28.8 (3.6)	30.2 (4.4)	30.8 (5.0)
Short physical performance battery (points) ^a^	8.4 (3.2)	10.7 (1.6)	10.2 (2.1)	8.4 (2.9)	7.9 (3.4)
Missing ^c^	6 (0.8)	0 (0.0)	0 (0.0)	1 (0.6)	5 (1.0)
Frailty trait scale (points) ^a^	38 (14.5)	23.6 (11.7)	28.9 (11.8)	37.7 (13.9)	40.9 (13.9)
Missing ^c^	22 (2.9)	0 (0.0)	0 (0.0)	4 (2.6)	18 (3.7)
Accelerometer wear time (min/valid day) ^a^	786.0 (82.6)	810.0 (84.3)	828.9 (80.1)	799.9 (81.5)	772.0 (79.6)
Sedentary time (min/valid day) ^a^	539.9 (90.6)	433.2 (46.7)	557.9 (67.4)	447.0 (65.1)	574.0 (76.1)
Light physical activity (min/valid day) ^a^	226.8 (86.2)	311.6 (50.1)	211.9 (44.0)	337.1 (58.4)	188.2 (64.5)
Moderate-to-vigorous physical activity (min/valid day) ^a^	19.4 (23.8)	65.2 (22.0)	59.1 (23.8)	15.8 (13.8)	9.8 (12.1)
≥ 10-min bouts of moderate-to-vigorous physical activity (min/day) ^b^	9.6 (17.7)	42.6 (18.2)	42.6 (22.3)	4.3 (6.1)	2.7 (5.1)
Meet WHO guidelines ^b^					
Yes	127 (16.5)	38 (100.0)	89 (100.0)	0 (0.0)	0 (0.0)
No	644 (83.5)	0 (0.0)	0 (0.0)	154 (100.0)	490 (100.0)
Light physical activity-to-sedentary time ratio ^a^	0.45 (0.24)	0.72 (0.11)	0.39 (0.09)	0.78 (0.26)	0.34 (0.13)

^a^Continuous variable; Mean (Standard Deviation)

^b^Categorical variable; n (Proportion (%))

^c^Missing data; n (%)

**Fig. 1:**
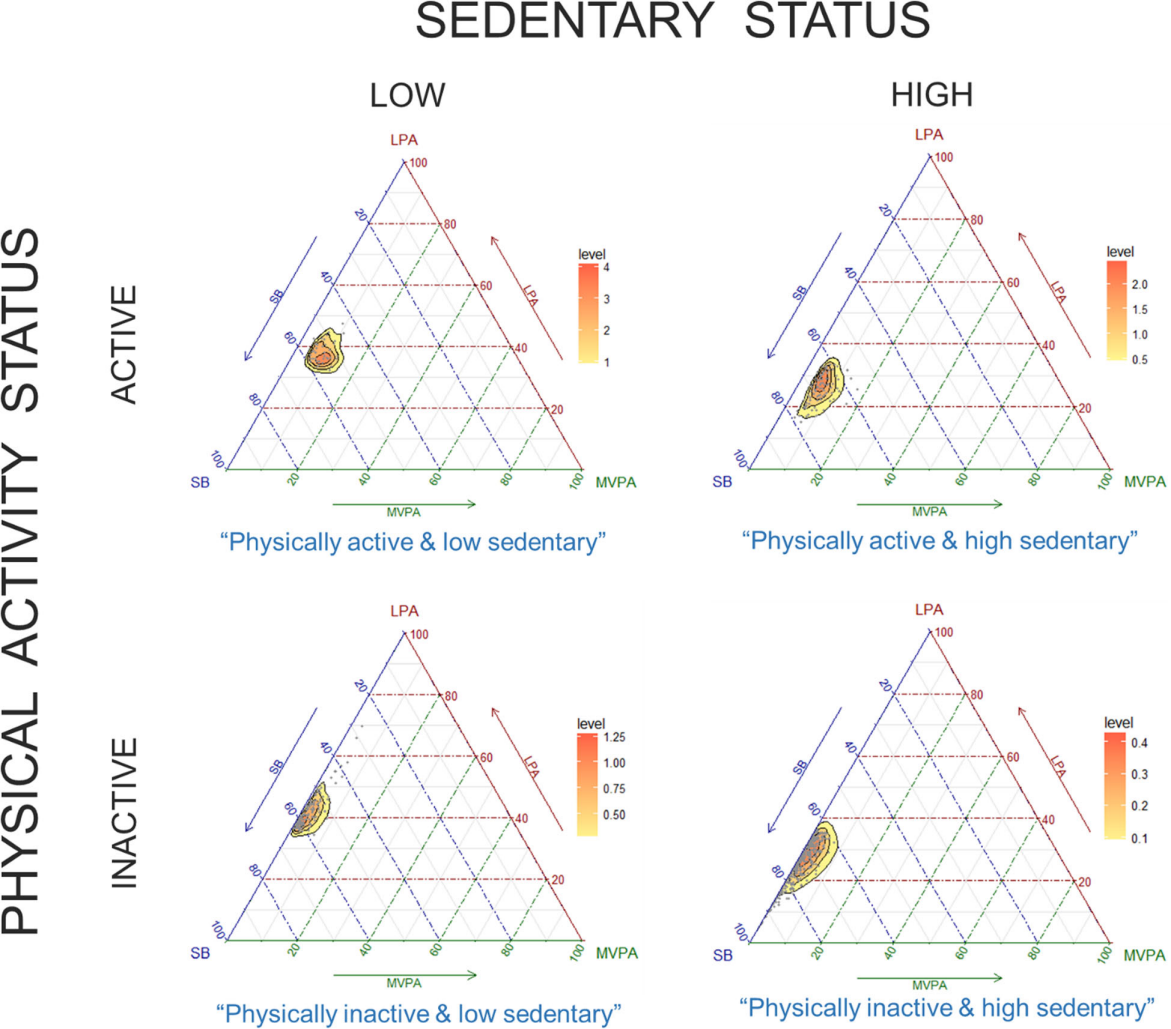
Ternary plots of the mutually exclusive behavioral categories of time spent in sedentary behavior (SB), light physical activity (LPA) and moderate-to-vigorous physical activity (MVPA). Low Sedentary: Quartile 1 of the ratio between the average light-intensity physical activity time and the average sedentary time. High Sedentary: Quartiles 2, 3 or 4 of the ratio between the average light-intensity physical activity time and the average sedentary time. Physically Active: ≥150 min of moderate-to-vigorous physical activity per week. Physically Inactive: < 150 min of moderate-to-vigorous physical activity per week. The overlapped heat map represents the distribution of the data points (the more intense the color the higher the concentration of data points)

The sample splits across the four different categories of movement as follows: [1] ‘physically active and low sedentary’: *n* = 38; 4.9%, [2] ‘physically active and high sedentary’: *n* = 89; 11.5%, [3] ‘physically inactive and low sedentary’: *n* = 154; 20.0%, and [4] ‘physically inactive and high sedentary’: *n* = 490; 63.6%. Ternary plots represent the time spent in each movement behaviour at a time for the different categories (Fig. 1).

Compared to ‘physically inactive and high sedentary’ participants, ‘physically active and low sedentary’ and ‘physically active and high sedentary’ individuals had significantly higher levels of physical functioning (β = 1.73; confidence interval [CI] = 0.77, 2.68; and β = 1.30; CI = 0.63, 1.98; respectively; *p* < 0.001) and lower frailty trait (β = − 13.96; CI = − 18.31, − 9.62; and β = − 8.71; CI = − 11.77, − 5.65; respectively; *p* < 0.001). Furthermore, ‘physically inactive and low sedentary’ group had significantly lower frailty score (β = − 2.50; CI = − 4.98, − 0.03; *p* < 0.05). However, differences on physical function between this two groups were not significant (β = 0.31; CI = − 0.23, 0.84; *p* = 0.26) (Table 2).

**Table 2:** Categorical associations with physical function and frailty (beta coefficients (95% CIs) and corresponding *p*-values)

Outcome	‘Physically active & low sedentary’		‘Physically active & high sedentary’		‘Physically inactive & low sedentary’		‘Physically inactive & high sedentary’
	Beta (95% CI)	*p*-value	Beta (95% CI)	*p*-value	Beta (95% CI)	*p*-value	
Short Physical PerformanceBattery (*n* = 765)	**1.73 (0.77, 2.68)**	**< 0.001**	**1.30 (0.63, 1.98)**	**< 0.001**	0.31 (−0.31, 0.84)	0.263	**Reference**
Frailty Trait Scale (*n* = 749)	**−13.96 (−18.31, −9.62)**	**< 0.001**	**−8.71 (−11.77, −5.65)**	**< 0.001**	**−2.50 (−4.98, −0.03)**	**0.047**	**Reference**

Adjusted linear regression models were fitted for physical function and frailty outcomes. The models were controlled for: age, sex, education, income and marital status

Bold indicates statistical significance at α = 0.05

Increased time spent in MVPA was significantly associated with higher levels of physical functioning (*p* < 0.001) and lower frailty trait (*p* < 0.001). Likewise, a higher light physical activity-to-sedentary time ratio was significantly associated with higher physical functioning score (*p* = 0.03) and lower frailty trait (*p* = 0.008) (Table 3).

**Table 3:** Continuous associations with physical function and frailty (beta coefficients (95% CIs) and corresponding *p*-values)

Outcome	Moderate-to-vigorous physical activity time		Light physical activity-to-sedentary behavior ratio	
	Beta (95% CI) ^a^	*p*-value	Beta (95% CI) ^b^	*p*-value
Short Physical Performance Battery (*n* = 765)	**0.03 (0.02, 0.04)**	**< 0.001**	**0.96 (0.09, 1.82)**	**0.030**
Frailty Trait Scale (n = 749)	**−0.18 (− 0.22, − 0.14)**	**< 0.001**	**−5.39 (− 9.34, − 1.44)**	**0.008**

Adjusted linear regression models were fitted for physical function and frailty outcomes. The models were controlled for: age, sex, education, income, marital status, moderate-to-vigorous physical activity time, light physical activity-to-sedentary time ratio, and accelerometer wear-time.

Bold indicates statistical significance at α = 0.05

^a^Beta coefficients represent a one minute increase in moderate-to-vigorous physical activity time per day

^b^Beta coefficients represent a one unit increase in the light physical activity-to-sedentary behavior ratio

## Discussion

The way in which time packed in a given day remains relevant for a wide range of health outcomes [32]. Previous research has identified the cardiometabolic [11] and mortality outcomes [13] of different movement patterns in adults and older adults, respectively. This is the first study assessing the associations of mutually exclusive categories of accelerometer-derived physical activity and sedentary time with physical function and frailty in older adults. The main findings were that participants who engaged in ≥150 min/week of MVPA had more favourable physical function and frailty profiles than those classified in the other movement pattern groups, regardless of sedentary status. Our results also suggest that engaging in more light intensity relative to sedentary time may have a positive impact on physical function and frailty status on the studied population, even in those individuals already meeting the physical activity guidelines. This might provide alternative intervention strategies to improve physical function and prevent frailty, as light activities are more feasible than more strenuous activity, particularly among previously inactive individuals.

Previous research have demonstrated that MVPA is effective to prevent, delay or even reverse functional limitations and frailty among older adults [33]. The present study provides novel data indicating that older adults who meet recommended physical activity levels, regardless of time spent in light-intensity activities relative to sedentary activities, have better physical function levels and frailty status compared to older adults who do not meet the required physical activity levels. These results emphasize the importance of engaging in sufficient MVPA, which could buffer some of the negative consequences of sedentary behaviour in preserving the physical functionality and reduce frailty in this population group [34, 35]. A recent meta-analysis involving more than 1 million adults has shown that engaging in higher amounts of strenuous activity can eliminate the mortality risk associated with too much sitting reported elsewhere [36]. The association of more intense activity with fitness levels partially explains why meeting the physical activity recommendations may overcome the harmful effects of sedentary behaviours. Thus, cardiovascular fitness has been proposed as a plausible mechanism mediating the relationship between sedentary behaviour and cardiometabolic health in older adults [37]. More studies are required to elucidate the role of fitness in the relationship between MVPA, sedentary behaviour, physical functioning and frailty in older adults.

Contemporary experimental [38, 39] and observational [40, 41] evidence emphasizes the health-enhancing role of light-intensity activities. In a recent meta-analysis by Chastin et al. [42], light-intensity physical activity emerged as potentially relevant for cardiometabolic health and mortality in adults and older adults, in particular among impaired individuals. Our estimates suggest that increasing the time in light physical activity relative to sedentary time has a positive impact on frailty levels in those considered physically inactive. Other studies have suggested the potential benefits of replacing sedentary behaviour with light-intensity physical activity to reduce frailty in older adults with multiple diseases [5]. It might be the case that in the more frail and functionally compromised individuals even small stimulus from light intensities can benefit their wider health [5]. Collectively, these findings are policy-relevant. Light-intensity physical activity is normally naturally embedded into the daily living of individuals (e.g. walking a dog, doing home chores or standing up while talking on the phone), therefore requiring no mental or physical effort or starting level to perform such activities, and thereby making light-intensity activities a pragmatic target for future public interventions to reduce frailty and improve physical function of older adults, particularly among those inactive (i.e. 83.5% in our sample) and that also depict very high levels of sedentary time (i.e. 63.6% in our sample) which might also be the most impaired individuals.

Interestingly, we identified the group meeting the physical activity guidelines (i.e. active) and showing higher levels of light intensity relative to sedentary time as the group with better frailty and physical function profile in our sample. Others have found similar results for cardiometabolic health [11] and mortality [13]. Recent epidemiologic evidence suggests that sitting time has deleterious cardiovascular and metabolic effects that are independent of whether or not adults meet the physical activity guidelines [31]. Our results suggest that engaging in more light-intensity activity relative to sedentary time beyond meeting the physical activity recommendations can provide with extra benefits in improving physical function and reducing frailty in older adults. Those individuals in our sample meeting the physical activity guidelines and engaging in more light-intensity activities extend their total volume of physical activity as supposed to those that meet the recommended amount of physical activity yet are sedentary, which could partially explain the extra benefit associated to that movement pattern [43]. Thus, promoting light-intensity activities could be a good approach to increase the total volume of physical activity and reduce sedentary time in those already meeting the physical activity guidelines, thereby enhancing their health, including increasing physical function and improving their frailty profile.

### Strengths and limitations

The present study has several strengths. First, the study includes a relatively-large sample of community-dwelling older adults with advanced age. Although there is no current established gold standard to determine physical function and frailty in older adults, the short physical function battery has positioned as one of the most used tools to objectively evaluate functional performance among older adults [44]. Similarly, the Frailty Trait Scale has been suggested as a more sensitive scale for detecting changes in the individual’s biological status than previously validated frailty instruments [16]. We also used accelerometer-measured procedures to assess physical activity and sedentary time.

Our study has also limitations. Firstly, the cut-off points used in the study to categorize the activity intensity of participants in the study can lead to a misclassification of both physical activity and sedentary time. However, the cut-off points used in this study are the most commonly reported in the literature for this age group [45], which make the results found here comparable with other investigations. Furthermore, ActiGraph devices are not able to discriminate between sitting and standing changes in the posture [46]. In order to obtain the activity status, bouts of at least 10 min were used, which may underestimate the time spent in MVPA. Nevertheless, further research is needed to consider the impact of the bout duration on frailty syndrome. Similar to what Bakrania et al. [11] reported, data could be overestimating the sedentary time [47], we therefore decided to use a more conservative approach for the extraction of sedentary status based on the behaviour of our population, an approach used in previous studies [11]. Loprinzi et al. defined *low sedentary status* as a positive light physical activity-to-sedentary time ratio [12]. If we had used the Loprinzi et al. [12] method, only 2.1% of our population would have been categorized as low sedentary status. This procedure used may have limitations and strengths. On the one hand, it is not influenced by the measurement of the accelerometer, but on the other hand, because is data-driven, may not be applicable to other populations. The use of this novel approach allows combining in mutually exclusive categories that best represent the different plausible combinations of physical activity and sedentary time within waking hours. Nonetheless, the cross-sectional nature of the research design used does not allow definitive conclusions to be drawn around the causal relationship between the outcomes of the study.

## Conclusions

We observed that physically active older adults had better physical function and frailty profiles than those considered physically inactive, even in the presence of high sedentary time. Higher levels of light-intensity physical activity relative to sedentary time seems to provide additional benefits in both physical function and frailty outcomes among those meeting the physical activity guidelines. Lower sedentary levels were associated with decreased frailty in physically inactive participants. Altogether, our findings reinforce the idea of the health-enhancing benefits of meeting the current physical activity guidelines. Also, our results highlight the relevance of light-intensity physical activity for inactive older adults. If our results remain experimentally true, light intensity physical activity can be promoted as a middle step intervention among inactive individuals to achieve the recommended levels of physical activity and improve their health. We should move beyond observational studies and confirm our results in well-design longitudinal, experimental studies.

## Data Availability

There is an established infrastructure, including a website (http://http://www.ciberfes.es/) and a review committee, through which data requests are handled. The hospital reviews and determines the purposes for the data requests and what data can be released. Data requests can be sent to: Research and teaching unit, Virgen del Valle Hospital Ctra. Cobisa S/N, 45071 Toledo – Spain, info@estudiotoledo.com.

## Abbreviations

FTS: Frailty Trait Scale
MVPA: Moderate-to-Vigorous Physical Activity
SPPB: Short Physical Performance Battery
TSHA: Toledo Study for Healthy Aging
WHO: World Health Organization
